# Functional Assessment of a New PBX1 Variant in a 46,XY Fetus with Severe Syndromic Difference of Sexual Development through CRISPR-Cas9 Gene Editing

**DOI:** 10.3390/genes14020273

**Published:** 2023-01-20

**Authors:** Laura Mary, Delphine Leclerc, Audrey Labalme, Pascale Bellaud, Séverine Mazaud-Guittot, Stéphane Dréano, Bertrand Evrard, Antoine Bigand, Aurélie Cauchoix, Philippe Loget, Anna Lokchine, Laurence Cluzeau, David Gilot, Marc-Antoine Belaud-Rotureau, Sylvie Jaillard

**Affiliations:** 1 CHU Rennes, Service de Cytogénétique et Biologie Cellulaire, F-35033 Rennes, France; 2 Inserm, EHESP, IRSET (Institut de Recherche en Santé, Environnement et Travail)-UMR_S 1085, Université Rennes 1, F-35033 Rennes, France; 3 Inserm U1242, Centre de Lutte Contre le Cancer Eugène Marquis, Université de Rennes, F-35033 Rennes, France; 4 Service de Génétique, Hospices Civils de Lyon, F-69007 Lyon, France; 5 CNRS, Inserm UMS Biosit, France BioImaging, Core Facility H2P2 Rennes, Université Rennes 1, F-35033 Rennes, France; 6 CNRS, UMR 6290 IGDR BIOSIT, Molecular Bases of Tumorigenesis, Université Rennes 1, F-35033 Rennes, France; 7 Service d’Anatomie Pathologique, Hôpital Pontchaillou, CHU Rennes, F-35033 Rennes, France

**Keywords:** DSD, sexual development, genome editing, RNA-seq, CRISPR-Cas9

## Abstract

Sexual development is a complex process relying on numerous genes. Disruptions in some of these genes are known to cause differences of sexual development (DSDs). Advances in genome sequencing allowed the discovery of new genes implicated in sexual development, such as PBX1. We present here a fetus with a new PBX1 NM_002585.3: c.320G>A,p.(Arg107Gln) variant, presenting with severe DSD along with renal and lung malformations. Using CRISPR-Cas9 gene editing on HEK293T cells, we generated a KD cell line for PBX1. The KD cell line showed reduced proliferation and adhesion properties compared with HEK293T cells. HEK293T and KD cells were then transfected plasmids coding either PBX1 WT or PBX1-320G>A (mutant). WT or mutant PBX1 overexpression rescued cell proliferation in both cell lines. RNA-seq analyses showed less than 30 differentially expressed genes, in ectopic mutant-PBX1-expressing cells compared with WT-PBX1. Among them, *U2AF1*, encoding a splicing factor subunit, is an interesting candidate. Overall, mutant PBX1 seems to have modest effects compared with WT PBX1 in our model. However, the recurrence of PBX1 Arg107 substitution in patients with closely related phenotypes calls for its impact in human diseases. Further functional studies are needed to explore its effects on cellular metabolism.

## 1. Introduction

The differences of sexual development (DSDs) are congenital variations characterised by a discrepancy between chromosomal, gonadal, and genital sex development. DSDs are classified in three categories according to the Chicago consensus [1]: sex chromosome DSDs, 46,XY DSDs, and 46,XX DSDs. In 46,XY DSDs, gonadal dysgenesis represents 1:10,000 births [2] and are characterised by disorders of testis formation and maintenance. The establishment of the bipotential gonad and its determination toward testis rely on complex signaling pathways. Briefly, the switch from a bipotential gonad to a testis relies on *SRY*, and its effector, *SOX9*. *NR5A1*, *WT1*, and *GATA4* also directly participate in *SRY* activation [3]. Other genes such as *DHH, DMRT1*, or *DAX1*, and more recently *DHX37* and *PPP2R3C*, also play a role in activating pathways leading to testis formation or repressing pro-ovarian signals [4,5]. Both copy number variants (CNVs) and a single nucleotidic variant (SNVs) have been found to impact these genes or their regulatory elements in patients presenting with isolated or syndromic 46,XY complete or partial gonadal dysgenesis. However, despite growing advances in deciphering the complexity of sex development, almost half of DSD patients does not have a molecular diagnosis [6]. Routine exome or genome sequencing (WES/WGS) combined with functional studies will help in highlighting new causes of DSDs. Focusing on syndromic DSDs may lead to the discovery of new ubiquitous pathways implicated in sexual development.

In this context, *PBX1* appears as a new gene implicated in syndromic DSDs. *PBX1* encodes a homeodomain transcription factor (TF), a protein of the TALE (three amino acid loop extension) TFs family. Its structure is composed of two domains (PBC-A and PBC-B) for dimerization with partner proteins (other TFs, from the TALE family as PKNOX or MEIS proteins, but also various other partners, such as HOX proteins, for example, [7]), and a homeodomain devoid to DNA binding. PBX1 needs heterodimerization with its partners to change conformation and to migrate to the nucleus. First described in rare forms of acute leukemia through the formation of a fusion transcript with E2A, PBX1 proved to be a protein implicated in various forms of cancer but also as a key factor of embryonic development [8]. Recently, *PBX1* deletions or SNVs have been implicated in the syndromic congenital anomalies of the kidneys and urinary tract (CAKUTHED), accompanied with various other developmental anomalies [9,10].

Until recently, *PBX1* variants were only reported in CAKUTHED patients. Interestingly, some patients (especially 46,XY individuals with *PBX1* missense mutations) presented with DSDs ranging from cryptorchidism and a micropenis to complete sex reversal, a phenotype that is not fully recalled by murine *PBX1*-KO models [7]. The role of PBX1 in sexual development is not yet fully understood. In mice, *Pbx1* KO affects gonadal development in both sexes, due to impaired cell proliferation, and XY mice are sex reversed [11,12]. Pbx1 acts downstream of Sf-1 (encoded by *Nr5a1*) in mouse gonads, but the link between these two proteins remains elusive. Moreover, *Pbx1*-KO animals present with Müllerian agenesis, a phenotype that recalls the uterine variations observed in *PBX1*-mutated patients [7]. Some authors suspected *WNT9B*, *HOXA10*, and *EMX2* were *PBX1* downstream effectors and were responsible for these Müllerian anomalies observed in patients [13,14].

**Figure 1:**
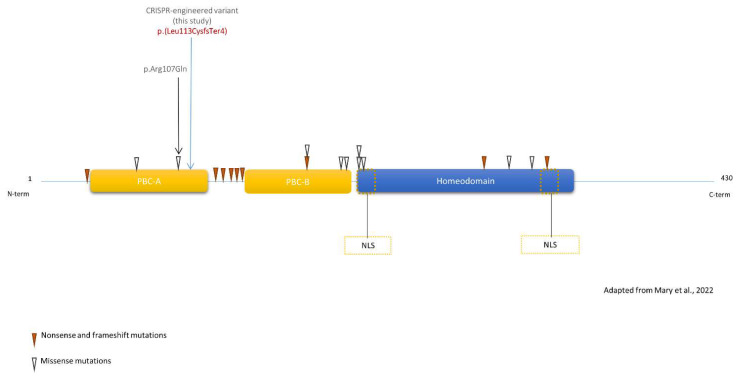
Schematic view of PBX1 with previously published variants. Blue arrow represents the CRISPR-engineered frameshift variant in clone KD2. Isoform: PBX1a - NM_002585.4 - NP_002576.1. All variants impact all PBX1 isoforms, except p.(Thr88Ile)(absent from NP_001340059). Yellow dotted boxes: Nuclear Localization Signal (NLS).

Two mutation hotspots were reported in *PBX1* patients: one between the two dimerization domains (PBC-A and PBC-B) and one in the homeodomain nuclear localization signal, this late hotspot containing two recurring variants, namely p.(Arg234Pro/Gln) and p.(Arg235Gln) (Figure 1). These variants have been explored through functional studies [15,16]. Both studies demonstrated impaired transactivation and dimerization properties in cellular models.

We report here the case of a stillborn fetus with a syndromic DSD phenotype carrying a substitution at *PBX1* Arg107 (NM_002585.3: c.320G>A, NP_002576.1:p.(Arg107Gln)). In this work, we assessed the functional impact of this variant by generating a cellular model knocked-down (KD) for *PBX1* and performing rescue experiments by transfection with *PBX1*-WT or *PBX1*-Arg107Gln coding plasmids.

## 2. Patient and Methods

### 2.1. Editorial Policies and Ethical Considerations

Written informed consent was obtained from the couple. All procedures were in accordance with the ethical standards of the Ethics Committee of Rennes University Hospital and the French law.

### 2.2. Patient

The patient was first addressed prenatally at 32 gestation weeks (GW) because of suspected isolated unilateral right kidney agenesis at ultrasound (US) examination. At 39 GW, US showed oligohydramnios and intrauterine growth retardation (IUGR). C-section was decided due to fetal arrhythmia. Apparent death was noticed at expulsion and the child could not be resuscitated. Standardized autopsy procedure was performed, including skeletal X-rays, macroscopic examinations, and histology analyses.

### 2.3. Cytogenetic Analyses

Chromosomal microarray (CMA) was performed using the Agilent Human Genome CGH microarray 180K, with a genome-wide median probe of 13 kb (Agilent Technologies, Santa Clara, CA, USA). The experiment was performed according to the manufacturer’s protocol. Patient’s gDNA were obtained from frozen tissues using a Prepito instrument (PerkinElmer Inc., Turku, Finland), or from cell culture as detailed below (Section 2.6). A graphic overview and analysis of the data were obtained using Agilent software. The probes were mapped using the GRCh37 genome version.

FISH analyses were performed on FFPE samples from both gonads using the Vysis CEP(X)/CEP(Y) commercial probe (Abbott, Abbott Park, IL, USA) targeting chromosomes X and Y centromeres, according to the manufacturer’s protocol.

### 2.4. Exome Sequencing and Bioinformatics Pipeline

Trio exome sequencing was performed at Lyon University Hospital. DNA was extracted from parental blood samples using the Hamilton automate machine and from frozen fetal tissue using a Prepito instrument (PerkinElmer). Exome DNA library was prepared with the MedExome preparation kit (Roche). High-throughput sequencing was performed on a NextSeq500 sequencer (Illumina). The BWA-MEM algorithm v.0.7 (Li & Durbin, 2009) was used to map the reads on the reference genome (GRCh37/hg19). The variant calling and variant predictions was performed according to GATK and FreeBayes best practices using a local pipeline (Papillyon). Variant prioritization was performed according to these predictions and information available on OMIM and ClinVar databases. Databases and in-silico prediction tools aggregators (Varsome, Mobidetails) were used to assess the pathogenicity of the variants.

### 2.5. Cell Culture

HEK293T (ATCC, further called “HEK cells” in this manuscript) cells were cultured on DMEM (Gibco) supplemented with 10% fetal bovine serum and 1% penicillin-streptomycin. All cells were maintained at 37 °C in a humidified incubator with 5% CO_2_.

### 2.6. Cell DNA Extraction, PCR and Sanger Sequencing

DNA was extracted from frozen cells pellets with the Nucleospin tissue DNA extraction kit (Macherey-Nagel). PCR was performed from 50 ng DNA with the Phusion™ High-Fidelity DNA Polymerase kit (Thermo Scientific). The list of PCR primers is present in Table S1. Sanger sequencing was performed according to standard protocols after PCR quality check on agarose gel.

### 2.7. RNA Extraction, Reverse Transcription and Quantitative PCR

RNA extraction was performed from frozen cells in culture plates or on frozen tissue samples (spleen, liver) by using the NucleoSpin RNA kit (Macherey-Nagel). Patient’s RNA quality was assessed using a Bioanalyzer 2100, Eukaryote Total RNA Nano chip (Agilent, Santa Clara, CA, USA). Quantitative PCR (qPCR) was performed after reverse transcription using the High-Capacity cDNA Reverse Transcription kit and the SYBR Green PCR Master Mix (Applied Biosystems), according to the manufacturer’s protocol. The primer sequences used are available in Supplementary Table S1. Raw data were extracted with QuantStudio Design and Analysis Software (Applied Biosystems) and 18S was used as a housekeeping gene for normalization.

### 2.8. Protein Extraction

Proteins were extracted from HEK cells with RIPA buffer (Thermo Scientific) supplemented with protease and phosphatase inhibitor. Protein quantification was performed with Pierce BCA protein assay kit (Thermo Scientific) and absorbance was measured at 562 nm on a microplate reader.

### 2.9. Western Blot

Protein samples were loaded in 4 to 12% Bis–Tris Gel (NuPAGE, Invitrogen) after denaturation and reduction according to the manufacturer’s protocol. Migration was performed for 1 h30, 200 V, 400 mA. After transfer and incubation with a blocking solution (1 h, room temperature, TBS-Tween 0.1%; 5% Bovine Serum Albumin), the membrane was tagged with the primary antibody overnight at 4 °C (rabbit anti-PBX1 antibody, HPA003505-Sigma-Aldrich, dilution 1/500, mouse anti-HSC70, #7298, Santa Cruz, dilution 1/2000) and then 1 h at room temperature with the HRP linked secondary antibody (HRP-linked anti-rabbit IgG, #7074 Santa Cruz, dilution 1/2000; HRP-linked anti-mouse IgG, #7076 Santa Cruz, dilution 1/2000). Uncropped Western blots are available in Supplementary Figure S1.

### 2.10. Methylen Blue Viability Assays

HEK were seeded in 96-well plates (15,000 cells/well). Approximately 24 h after seeding, cells were used for determining viability threshold for either puromycin treatment, PBX1 plasmid, or siRNA transfections. For puromycin viability assays, cells were treated with increasing puromycin concentrations ranging from 0.1 ng/µL to 16 ng/µL in 100 µL of medium/well. For plasmid viability assays, cells were transfected with either PBX1a-WT or PBX1a-320G>A plasmid concentrations ranging from 0.1 ng/µL to 0.5 ng/µL in a solution of OptiMEM medium (Gibco) and 2% of Lipofectamine 2000 (ThermoFisher Scientific). For siRNA viability assays, cells were transfected with either control or PBX1 siRNA concentrations ranging from 5 nM to 100 nM in a solution of OptiMEM medium (Gibco) and 2% of Lipofectamine RNAimax (ThermoFisher Scientific). After 2 days of treatment, cells were rinsed, fixed in 95% ethanol for 30 min and dried. Methylen blue was added to the cell pellet for 30 min. The plate was then washed and dried, and the cell pellets were suspended in HCl. Absorbance was measured with microplate reader at 620 nm.

### 2.11. siRNA Transfection

HEK were seeded in 6-well plates (500,000 cells/well). Approximately 24 h after seeding, cells were transfected with 25 nm siRNA CTRL (#230285603, IDT) or siRNA *PBX1* (IDT, sequence available in Table S1) with Lipofectamine RNAimax (ThermoFisher Scientific) according to the manufacturer’s instructions.

### 2.12. Cloning

Single guide RNAs (sgRNA) were designed according to the recommendations of [17], Table S1. BPK1520 backbone (Addgene plasmid #65777) was used for integration of sgRNAs DNA sequence by golden gate assembly, as previously described [18]. Plasmids were transformed into NEB^®^ Stable competent *E. coli* C3040H (New England BioLabs) by following the manufacturers’ recommendations. Bacteria were cultured according to [19]. Plasmid DNA was purified with NucleoBond^®^ Xtra Maxi kit (Macherey Nagel). Sanger sequencing was performed for construct validation.

### 2.13. CRISPR-KD

HEK were seeded in 6-well plates (500,000 cells/well). General protocol for CRISPR transfection and selection was described elsewhere [19]. Transfection was performed using a mix of pSpCas9(BB)-2A-Puro plasmid (PX459#62988, Addgene, gift from F. Zhang, [19]), plasmid encoding the sgRNA (see “Cloning” section) and a control plasmid encoding GFP (PMirZIP #25037, Addgene). Three different sgRNA were tested. Transfections with the empty BPK1520 backbone + PMirZIP +/- pSpCas9(BB)-2A-Puro plasmids were used as negative controls. Puromycin was used at a concentration of 8µg/mL (determined through methylene blue viability studies, see above) to select Cas9-puro-transfected cells. Limit dilution of transfected cells was performed as described by [19]. Wells containing only one clonal colony after 2–3 weeks of culture were harvested and amplified in 24-well plates. When confluency was reached, DNA from each clone was extracted for Sanger sequencing (see above) to determine the clone’s genotype. Clones showing frameshift variations located at the sgRNA’s target sequence (Supplementary Figure S2) were amplified in 6-well plates in duplicates, and PBX1 synthesis defect were checked through Western blot (see above). Clones presenting with both frameshift variants and lower PBX1 signals at Western blot compared to a positive control were used for rescue studies and functional analyses (proliferation studies, qPCR, RNA-seq).

### 2.14. Site-Directed Mutagenesis on PBX1 Plasmid

*PBX1a*-coding plasmid was obtained from Addgene (*PBX1a*-pCMV1 #21029, Addgene, gift from Corey Largman) and PBX1a exon 3 sequence was checked by Sanger sequencing. Site-directed mutagenesis was performed using Agilent Stratagene QuickChange XL kit (Supplementary Table S1). The reaction product was used to transform XL1-Blue supercompetent cells according to the manufacturer’s protocol. Bacteria seeding and amplification, plasmid extraction, and sequencing were performed as described in the “Cloning” section.

### 2.15. Proliferation Studies

HEK cells and CRISPR-KD-modified cells were seeded in 6-well plates (300,000 cells/well) or in 96-well plates (20,000 cells/well) and transfected with either PMiR-ZIP and PBX1a-WT or PMiR-ZIP and PBX1a-320G>A plasmid as previously described. Untransfected cells were used as negative controls. Cell proliferation was studied after 3 days, either through methylene blue treatment (see above) for 96-well plates (negative controls and transfected cells), or through Malassez counting chamber after digestion with TrypLE™ Express enzyme (1X) and resuspension in DMEM, for 6-well plates (negative controls only). Before cell counting on 6-well plates, pictures at ×10 and ×20 magnification were captured using an Evos M5000 microscope (Invitrogen).

### 2.16. RNA-seq

RNA-seq was performed at BGI Tech (Shenzhen, China). RNA was extracted from WT HEK or KD clone after 3 days of transfection with either PBX1a-WT or PBX1a-320G>A plasmids. Transcriptome library was performed with the DNB preparation kit (BGI). High-throughput paired-end sequencing was performed on a DNBSEQ-G400 sequencer (BGI). The HISAT2 algorithm [20] was used to map the reads on the reference genome (GRCh38/hg38). Sequencing data filtering was performed using SOAPnuke algorithm [21]. Bowtie2 [22] and RSEM [23] protocol was then used to align the reads to the reference genes and quantify gene expression. Differential gene expression was estimated using the DEGseq method [24] and gene annotation was performed with GO and KEGG enrichment analyses. Differential alternative splicing and fusion gene analyses were studied using rMATS statistical model [25] and Ericscript [26], respectively. Significance thresholds for differentially expressed genes were set as follows: |log2FC| ≥ 1 and False Discovery rate (FDR) ≤ 0.01 according to Poisson distribution and |log2FC| ≥ 0 and q-value ≤ 0.05 according to DEGseq2.

## 3. Results

### 3.1. Patient

**Figure 2:**
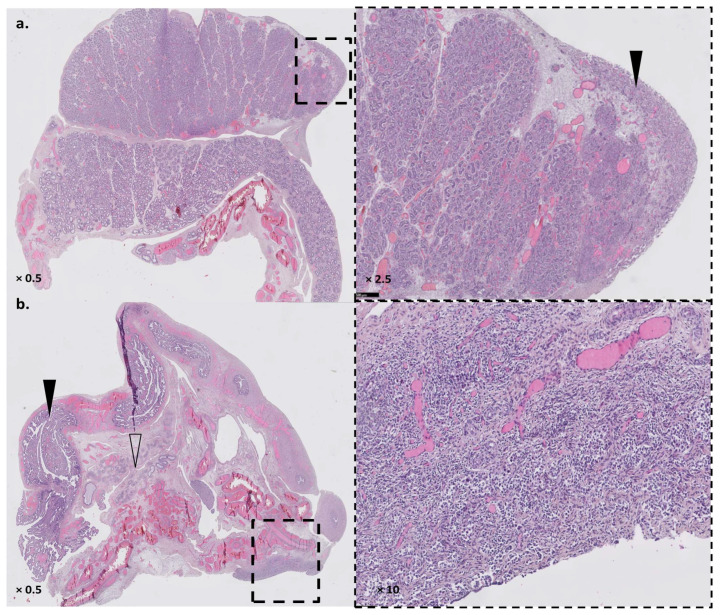
Gonadal phenotype of the fetus. Panel (**a**): right testis-like gonad with small area of undifferentiated tissue (dashed box, black arrowhead). Panel (**b**): left streak gonad (dashed box) with both fallopian tube (black arrowhead) and epididymis (white arrowhead). Magnification shows an undifferentiated ovarian-like tissue with rare primordial germ cells and absent albuginea.

The patient was a deceased neonate presenting with a complex DSD with prominent genital tubercle, a urethral meatus opening at the base of the genital tubercle, unfused lateral genital folds without a gonad inside, and an absent vaginal opening. Autopsy found a small bicornuate and septated uterus connected to the first tier of a rudimentary vagina, and two pelvic gonads. The right gonad had a testis-like gross appearance. Microscopic analyses found a testis-like gonad with focal dysplasia, the presence of Leydig cells, and an epididymis (Figure 2a). The left gonad was an undifferentiated streak gonad accompanied by both a fallopian tube and an epididymis (Figure 2b). The child also presented with major lung hypoplasia (lung weight/fetal weight < 0.006 for normal values > 0.012 for fetuses > 26 GW) that could not be fully explained by late oligohydramnios, and which led to neonatal death. The autopsy did not confirm the unilateral renal agenesis but highlighted a misplaced right kidney (pelvic) and a normally placed left kidney. Both renal pelvises were anteriorly rotated. Both kidneys were hypoplastic, albeit histologically mature. X-rays highlighted a missing rib pair. Growth and histological examinations were otherwise normal, showing only non-specific hypoxic lesions.

CMA did not reveal any chromosomal imbalances associated with the phenotype and found a XY gonosomal sex. FISH on both gonads highlighted only XY cells, dismissing a gonadal dysgenesis due to XX/XY mosaicism. Trio exome sequencing revealed a heterozygous de novo *PBX1* variant: chr1:g.164761785G>A, NM_002585.3:c.320G>A, p.(Arg107Gln). The variant was predicted to be pathogenic by most of the in silico tools used. The nucleotide position was strongly conserved and variants in this region are not reported in general population databases. No other pathogenic or likely pathogenic variants in mendeliome genes were found.

### 3.2. Generation of a Cellular Model Knocked-Down (KD) for PBX1, with Impaired Proliferation and Support Adhesion

**Figure 3:**
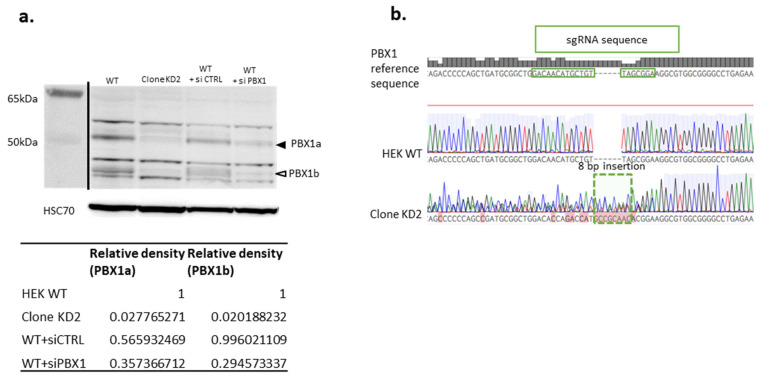
Obtention of a *PBX1* KD clone. Panel (**a**): Western Blot demonstrating the diminution of both PBX1a (black arrowhead) and PBX1b (white arrowhead) expression PBX1 KD clone (clone KD2). WT HEK cells transfected with control siRNA (WT + si CTRL) or PBX1 siRNA (WT + si PBX1) served as negative and positive controls, respectively. WT: non-transfected HEK cells. Panel (**b**): Sequence of *PBX1* exon 3 in WT HEK cells (HEK WT) and PBX1 KD clone (clone KD2) showing an 8-bp insertion (dashed box) at the sgRNA’s target sequence (green box) in KD2. Uncropped Western Blot is presented in Supplemental Data (Figure S10).

Due to the delay between the child’s death and sample collection, no cultured cells from the patient could be obtained. RNA extractions on multiple frozen tissues (liver, thymus, spleen) only generated degraded RNA that could not be used for further explorations (RNA Integrity Number between 1.9 and 2). We used HEK cell line in order to generate clonal cell lines KO for *PBX1* through CRISPR-KD genome editing. Three single guide RNA (sgRNA_1 to 3) were used in the experiments. After CRISPR editing, limit dilution, and 2–3 weeks of culture, 32, 27, and 13 clones were obtained for each sgRNA, respectively. All were amplified and Western Blot (WB) was performed to determine the edited clones. No complete abolition of PBX1 expression (i.e., KO clones) was observed (Figure 3a and Supplementary Figure S1). Clones with diminished PBX1 expression were then Sanger-sequenced at the sgRNA’s target sequence to assess their genotype (Figure 3b and Supplementary Figure S2). siRNA against *PBX1* was used to validate the WB results (Figure 3a). One clone (clone KD2) with a clear PBX1-diminished signal (Figure 3) was retained for further analyses. PCR-sequencing showed an 8-bp insertion in clone KD2, responsible from a frameshift and a premature stop codon PBX1(NM_002585.4):c.337_338insGCCGCAAC, (p.Leu113CysfsTer4), suggesting nonsense-mediated decay (NMD) as a mechanism explaining the KD. Sanger sequencing showed a residual detection of WT *PBX1* exon 3 sequence, suggesting that one of the three *PBX1* alleles (HEK293T are pseudo-triploid) was non-edited. This explained the residual PBX1 expression at Western Blot.

**Figure 4:**
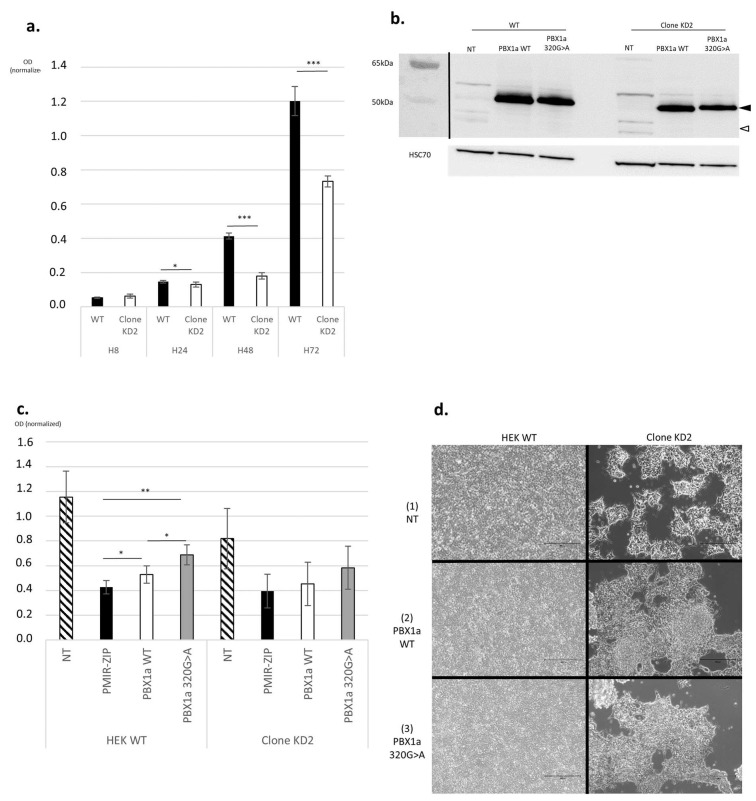
PBX1 partial LoF impairs cell proliferation, not fully rescued by *PBX1a* transfection. (**a**) *PBX1* LoF impairs cell proliferation (Methylen blue test). OD: optical density. H: hours. Bars represent standard deviations (SD). (**b**) WB assessing PBX1a overexpression (black arrowhead) in transfected HEK WT cells (HEK WT) and KD2 clones. White arrowhead: PBX1b. (**c**) Transfection with either PBX1a WT or 320G>A plasmids enhances proliferation in WT HEK cells in comparison with control (PMiR-ZIP, a GFP-encoding plasmid), but not in *PBX1* KD cells, after 3 days of culture. Bars represent SD. (**d**) *PBX1* KD clones are unable to grow as a monolayer and reach confluency, a phenotype slightly rescued by both WT and mutant PBX1a overexpression. For each quantitative experiment, 3 biological replicates were realized. Each biological replicate comprised 3 technical replicates per sample. * = *t* test between 0.01 and 0.05, ** = *t* test between 0.001, and 0.01, *** = *t* test between 0.0001 and 0.001.

Compared with WT HEK cells, clone KD2 showed significant proliferation impairment (Figure 4a and Supplementary Figure S3) and grew as colonies instead of growing as a monolayer (Figure 4d, panel (1)). Clones KD2 detached from the flask bottom as soon as 3 days of culture and before reaching confluency, suggesting cell–cell and cell–support adhesion anomalies. aCGH results showed a few differential imbalances between clone KD2 and WT cells (Figure S11).

### 3.3. Rescue Experiments by Transfection with PBX1a-WT or PBX1a 320G>A Plasmids

Both WT HEK (further called WT HEK) cells and clone KD2 cells were transfected with a plasmid encoding either WT *PBX1a* or the *PBX1a* 320G>A (further named “mutant PBX1a”) variant of interest.

Transfection efficiency was assessed through WB and both *PBX1*-coding plasmids led to PBX1 overexpression in all cell types (Figure 4b and Supplementary Figure S10).

#### 3.3.1. PBX1 Overexpression Enhances Cell Proliferation

Transfection with WT *PBX1a* plasmid significantly enhanced cell proliferation in WT HEK compared with a transfection control (Figure 4c). Transfection of mutant *PBX1a* also significantly increased cell proliferation compared with control. This proliferation enhancement through the mutant *PBX1a* transfection was also significant compared with WT *PBX1a* transfection. A similar pattern of proliferation enhancement was also observed in clone KD2 with both WT and mutant PBX1a plasmids, albeit non-significantly (Figure 4c).

#### 3.3.2. Overexpression of PBX1a Slightly Rescues KD2 Normal Adhesion

Non-transfected KD2 cells show impaired cell–cell and cell–support adhesion capacities (Figure 4d, panel (1)). Transfection with either WT or mutant PBX1a slightly rescued the adhesion phenotype of KD2 cells, with larger and thinner clusters of cells (Figure 4d, panels (2) and (3)). However, early detachment was still observed before confluency. On the contrary, transfection with WT or mutant PBX1a plasmid in WT HEK cells did not modify their adhesion phenotype (Figure 4d, panels (2) and (3)).

#### 3.3.3. Targeted RT-qPCR Suggests MEIS1 as a Potential Disrupted Gene

RT-qPCR was then performed to explore *PBX1* target genes modulations in both cell types. Target genes (*CCND1*, *MEOX1*, *FZD2*, *ZHX2*, *AR, NFE2L1*) were chosen according to [27,28] and depending on their expression profile in WT HEK cells (based on Human Protein Atlas data). PBX1 cofactors MEIS1 and PKNOX1 were also studied. For each cell type, WT *PBX1a* plasmid transfection was considered as a control condition. PBX1 cofactor MEIS1 was less expressed in WT HEK cells transfected with mutant *PBX1a*, while expression was similar in all transfected KD2 cells. Other gene expressions were not significantly modified (Supplementary Figure S4).

### 3.4. RNA-seq

To further assess RT-qPCR preliminary results, we performed RNA-seq on WT HEK and clone KD2 transfected with either WT or mutant PBX1a plasmids. About 44 million 100 bp-long paired-end reads per sample were generated. The average mapping ratio with reference genome was 96.14%, the average mapping ratio with gene was 78.27%, 16654 mRNA-coding genes were identified.

#### 3.4.1. Both Cell Lines Expressed Multiple PBX1 Transcripts

**Figure 5:**
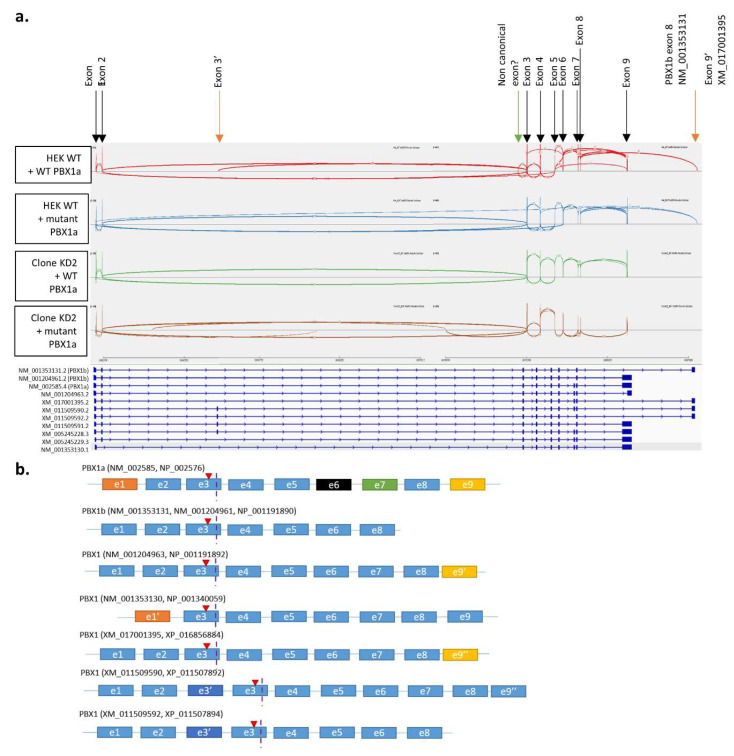
*PBX1* transcripts in HEK cells and clone KD2. (**a**) Sashimi plot of *PBX1* in HEK cells + WT PBX1a (1), HEK cells + PBX1a-320G>A (2), clone KD2 + WT PBX1a (3), and clone KD2 + PBX1a-320G>A. Exon junction coverages < 5 are hidden for lisibility. Black arrows represent exons of *PBX1a* isoform (NM_002585). (**b**) Schematic view of *PBX1* transcripts. Red triangle represents the location of the c.320G>A mutation. Vertical dotted purple line represents the location of the CRISPR-generated frameshift insertion.

Focusing first on PBX1 expression, we showed that it was expressed at high levels in all four samples, albeit slightly significantly less expressed in KD2 cells compared with WT HEK cells (Supplementary Figure S5). The variation c.320G>A was found in all *PBX1* transcripts in cells transfected with mutant *PBX1a* plasmid (Supplementary Figure S7). Both WT HEK cells and clone KD2 cells expressed a PBX1a transcript. Transcript XM_017001395, which was close to, but not identical to, *PBX1a* (use of an alternate exon 9) was also highly expressed. However, the alternate exon 9 has a sequence closely related to *PBX1a* exon 9 and XM_017001395 represents a predicted transcript, which was not assessed through previous functional analyses. Moreover, the alternate exon 9 sequence was absent from our plasmid. This late exon 9 was poorly covered (<20 reads per sample). Thus, it may represent falsely aligned *PBX1a* transcripts. *PBX1b* transcript (NM_001204961) was slightly expressed in both cell lines, albeit significantly less in clone KD2. HEK cells produce at least four other *PBX1* transcripts (Figure 5, Table 1), including a transcript with an alternative exon 9, which was absent from KD2 cells.

#### 3.4.2. Gene Expression between WT HEK and KD2 Cell Lines Remains Different despite Being Transfected with the Same Plasmid

**Figure 6:**
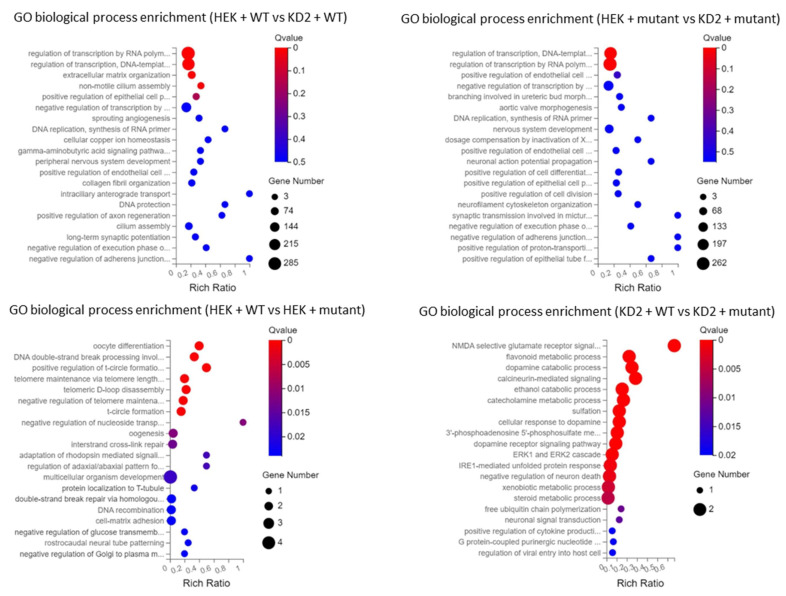
Bubble charts of GO biological processes enrichment in each comparison group. HEK + WT/mutant: WT HEK cells transfected with WT or mutant *PBX1a* plasmid. Clone KD2 + WT/mutant: KD2 cells transfected with WT or mutant *PBX1a* plasmid.

RNA-seq results are summarized in Supplementary Table S2. Scatter plots showing DEGs between each comparison group are given in Figure 6.

When we compared mutant *PBX1a*-transfected cells to WT *PBX1a*-transfected cells (control vs. case analysis), only one gene (U2 small nuclear RNA auxiliary factor 1, *U2AF1*) was significantly downregulated in mutants (log2 fold change between case and control: −23.45). The WT-transfected cells and the mutant-transfected showed in fact strong differences on the PCA plot, explaining the fact that only one gene was significantly deregulated when grouping cells in control vs. case analyses (Supplementary Figure S6). *U2AF1* was only expressed in WT *PBX1a*-transfected HEK cells (TPM at 56.94 for WT-transfected WT HEK cells and was null for the other three samples, FDR ^(Clone KD2_WT/Hek_WT)^ = 1.33 × 10^−116^ and FDR ^(Hek_MUT/Hek_WT)^ = 7.46 × 10^−120^).

**Figure 7:**
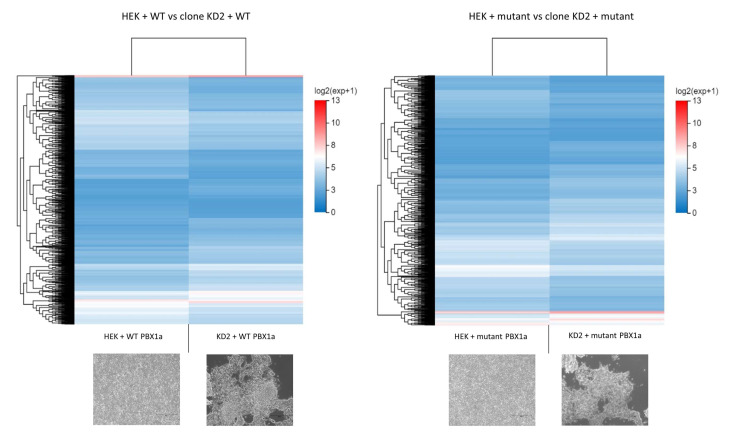
Heatmaps comparing the differential expression between WT HEK and *PBX1* KD cells transfected with similar *PBX1* plasmids. HEK + WT/mutant: WT HEK cells transfected with WT or mutant *PBX1a* plasmid. Clone KD2 + WT/mutant: KD2 cells transfected with WT or mutant *PBX1a* plasmid.

To assess this result, we first explored the differentially expressed genes (DEGs) between WT HEK cells and KD2 cells transfected with WT *PBX1a* plasmid. We found 2118 DEGs between these two groups (1219 downregulated genes and 899 upregulated genes). The most significantly down-regulated biological processes in KD2 cells (q-value ≤ 0.05) according to GO and KEGG classifications were the regulation of transcription (more than 200 genes, including *EMX1*, *GATA4*, *SOX8*, *AR*, *HOXA4*, *HOXA11*, *HOXB5*, and *HOXD4*, *MYC*, and *SMAD* genes) and non-motile cilium assembly (18 genes, including *BBS1*, *CEP290*, and *PCM1*). Upregulated functions included extracellular matrix organization (55 genes, including collagens I to VI subunits, *CCN1* and *ICAM* genes), and plasma membrane repair. DEGs between cell lines transfected with mutant PBX1a plasmid were similar (1985 DEGs), but the only significantly downregulated cellular processes were the regulation of transcription (more than 200 downregulated genes in KD2 cells, including all the abovementioned genes and *SOHLH2*). Plasma membrane repair genes also remained upregulated (Figure 6 and Figure 7). Thus, WT HEK and clone KD2 are highly different, a difference in transcriptome that remained whatever the plasmid transfected. Such a difference explains the difficulties to find DEGs in a control vs. case analysis. These differences may in fact reflect the consequences of the CRISPR-engineered frameshift variant in KD2 cells.

#### 3.4.3. DEGs between WT and Mutant *PBX1a* Transfections Depend on the Cell Line

**Figure 8:**
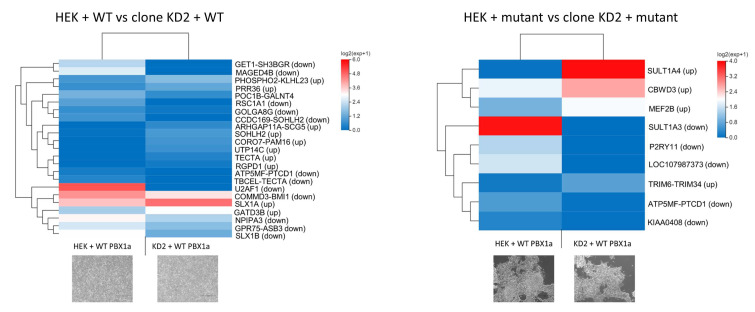
Heatmaps comparing the differential expression between a cell line transfected with WT or mutant PBX1 plasmids. HEK + WT/mutant: WT HEK cells transfected with WT or mutant PBX1a plasmid. Clone KD2 + WT/mutant: KD2 cells transfected with WT or mutant PBX1a plasmid. Up/down: the gene is up/down regulated in cells transfected with mutant plasmid comparing with cells transfected with WT plasmid.

We then studied DEGs between WT- and mutant-transfected cells without clustering both cell types (Figure 8). We only found a few DEGs in WT vs. mutant experiments. In total, 23 DEGs were identified for HEK cells, implicated in various cellular functions according to GO (oocyte differentiation, DNA double strand break repair, telomere maintenance, regulation of nucleoside transport, Figure 6 and Figure 8). Ten DEGs were identified in KD2 cells, impacting dopamine and steroid metabolism and cytokine production. In such analysis, only the *ATP5MF-PTCD1* gene was downregulated in both cell types.

These DEGs differed from preliminary RT-qPCR results. *CCND1* and *AR* were upregulated in WT *PBX1a*-transfected KD2 cells vs. WT *PBX1a*-transfected HEK cells (FDR 8.4 × 10^−78^ for *CCND1* and 8.4 × 10^−81^ for *AR*). *AR* was also upregulated in mutant *PBX1a*-transfected KD2 cells vs. WT *PBX1a*-transfected KD2 cells (FDR 1.4 × 10^−71^). *MEIS1* expression was not significantly deregulated.

#### 3.4.4. Transfection with Mutant PBX1a May Alter Skipped Exon Splicing Events

Since *U2AF1*, which encodes for a subunit of the splicing factor U2AF that recognizes the pyrimidine-rich tract present at metazoan 3-prime splice sites, was predicted to be downregulated in mutant-transfected cells compared with WT-transfected ones, we then focused on differential alternative splicing events (DASE) among groups. Transfection of mutant plasmid did not significantly modify the splicing events compared with WT plasmid, except in clone KD2, where a diminution of skipped exons events was observed. On the contrary, we observed a large number of DASE when comparing WT HEK cells and clone KD2, with a dramatic diminution of all splicing events in clone KD2 cells compared with WT HEK cells regardless of the plasmid transfected. Most of these late DASE were skipped exons (Supplementary Figure S8).

## 4. Discussion

### 4.1. PBX1 Is a DSD Gene

Recently, Arts et al. [29] reported two stillbirth siblings of both chromosomal sexes presenting with the most severe sides of *PBX1*-linked developmental diseases: CAKUTHED, diaphragmatic eventration, lung hypoplasia, and sex reversal in the 46,XY fetus. Both fetuses carried a new PBX1 NP_002576.1:p.(Arg107Trp) variant. These phenotypes strongly recall the post-mortem findings observed in our patient, who also carried a similar substitution at the arginine 107. PBC-A variants are rare, and the absence of functional explorations for Arg107 missense variants prompted us to create an in vitro model expressing PBX1 proteins with Arg107Gln substitution.

### 4.2. Partial PBX1 Loss of Function (LoF) Impairs Cell Proliferation

We first aimed at generating a HEK cellular model KO for *PBX1* through CRISPR-Cas9 editing, but we obtained only a few viable KD clones and no KO clones. The impossibility of generating KO clones suggests that *PBX1* KO is lethal for HEK cells, as has been previously observed in murine models [12]. *PBX1* KD clones presented with a distinct phenotype from WT cells, with a decreased proliferation rate and a distinct growth phenotype, suggesting abnormal cell adhesion and intercellular communication. KO for Pbx1 in mouse models are always embryonically lethal and the embryos presented with defects in nephrogenic mesenchyme proliferation [12]. In a large set of cancer types (lymphoblastic leukemia, lung adenocarcinomas and squamous cancers, hepatocellular, and bladder cancers, etc.), *PBX1* amplification promotes cell proliferation and metastasis [30]. The introduction of a missense mutation in *PBX1* is sufficient to avoid tumorigenesis in gastric cancer cells [31]. Since HEK cells are derived from immortalized human embryonic kidney cells, we suggest that complete loss of *PBX1* expression is always lethal for this cell line. *PBX1* partial LoF is sufficient to induce defects in HEK cells proliferation.

### 4.3. Transfection with PBX1-Coding Plasmids Does Not Fully Rescue a PBX1-KD Phenotype

Transfection with plasmids containing either WT or 320G>A *PBX1a* isoform led to a significantly increased proliferation rate in WT HEK cells but not in KD cells, despite the fact that both mutant and WT plasmids were expressed at high levels in both cell lines. Cellular adhesion defects were only partially rescued in KD cells, whatever the plasmid transfected. The example of *U2AF1* showed that at least some gene expressions were not rescued by the WT *PBX1a* transfection in clone KD2. At the RNA-level, transfection with the same plasmid led to significant DEGs between WT and KD cells. Thus, contrary to what we expected, transfection of the WT *PBX1* plasmid did not lead to the reappearance of a “WT-like” phenotype in *PBX1*-KD cells, and the expression of a mutant PBX1 protein did not impair cell proliferation in HEK cells. We suggest some hypotheses to explain these results. First, WT HEK cells expressed higher levels of *PBX1* transcripts contrary to KD cells, including specific isoforms absent from KD cells. PBX proteins, including PBX1, are sensitive to intracellular concentration [32]. Mutated PBX1 proteins also have dominant-negative effects and are able to abolish proliferation in a cancer cell line [33]. Diminished intracellular concentration of PBX1 associated with the production of some truncated PBX1 proteins in KD clones may explain the absence of full rescue in this cell line. On the contrary, higher PBX1 concentrations, even mutant proteins, may be sufficient to induce proliferation in WT HEK cells. Overall, only a few DEGs were observed between WT- and mutant-*PBX1a* plasmid transfection experiments, despite the fact that a large amount of PBX1 proteins was produced in all cell lines. We can also suggest that co-transfection with a plasmid encoding a PBX1 cofactor (PKNOX1 for example) would have amplified the effects of *PBX1* plasmid transfection [15]. In fact, PBX1 is unable to act alone and needs heterodimerization with one (or more) cofactor(s) to reach the nucleus and modify its target genes’ expression. Level-up PBX1 concentration in cells expressing normal amounts of cofactors may lead to a subset of inactive PBX1 monomers due to insufficient cofactor concentrations. These findings suggest that the *PBX1*-320G>A variant only has mild effects in HEK cells.

Nevertheless, DEGs between WT HEK cells and KD cells shed the light on interesting PBX1 target genes. Numerous *HOX* genes, *GATA4*, *SOX8*, *AR*, *MYC*, and *SMAD* genes showed differential expression between WT and KD cell lines. *MYC* is a strong proto-oncogene controlling cell cycle and cell growth [33], its downregulation probably playing a role in the decreased KD cells proliferation capacities. Other DEGs could sum up, at least partially, the phenotype observed in *PBX1*-mutated patients. *HOX* genes, notably *Hoxa11* in mice, are responsible for the establishment and segmentation of the Müllerian ducts, precursors of the uterus and fallopian tubes [34]. Through *GATA4*, *PBX1* controls cardiomyocyte precursors’ proliferation and maturation [35]. SMAD proteins, especially SMAD3 and SMAD6, also play a role in the regulation of endocardial cushion transformation [36,37]. SOX8, a transcription factor showing redundant functions with SOX9, was suggested as responsible for 46,XY DSD [38]. *AR* encodes for the androgen receptor and is responsible for the partial to complete androgen insensitivity in 46,XY patients, a syndrome that may lead to normal-appearing female external genitalia.

Despite the moderate effect of *PBX1a*-320G>A plasmid transfection, some interesting questions arose from our results. *U2AF1*, encoding one of the subunits from the splicing factor U2AF, was downregulated in mutant-transfected WT HEK cells. This splicing factor plays an important role in targeting 3′ splicing sites and in intron removal [39]. Mutations in this gene increases the risk of progression from myelodysplasia to secondary acute myeloid leukemia, through splicing dysregulation [40]. WT HEK cells transfected with the mutant PBX1a plasmid also demonstrated upregulation of *SLX1A* and downregulation of SLX1B. These proteins represent the catalytic subunit of the *SLX1*-*SLX4* structure-specific endonuclease. This complex is required for the repair of specific types of DNA lesions and is critical for cellular responses to replication fork failure [41]. KD cells transfected with *PBX1a* mutant plasmid showed upregulation of a read-through transcript, *TRIM6-TRIM34*, which is considered as a splice variant of *TRIM34. TRIM34* localizes to the mitochondria and mediates apoptosis through the mitochondrial pathway [42]. Thus, the expression of *PBX1a*-320G>A transcript may lead to splicing defects (albeit moderately observed in our experiments) and increased genome instability through abnormal resolution of Holliday junctions. *PBX1a*-320G>A transcript may also modify cell proliferation through an increase in *TRIM6-TRIM34* transcript. Finally, most of the DEGs observed between transfected cells are read-through transcripts, i.e., transcripts arising from inefficient 3’-end cleavage of nascent mRNAs, with the production of an mRNA containing two contiguous genes. Such phenomenon can be observed in cells under biological stress such as transfection, but also in several disease contexts including viral infections and renal cancer [43]. Some of these transcripts are known to be biologically active (such as *TRIM6-TRIM34*) but the precise role of such “fusion” transcripts remains unclear for now. Moreover, they are suspected to be overlooked in RNA-seq studies, due to the incorrect sequence assignation to one of the two parent genes [43]. Interestingly, KD cells transfected with mutant *PBX1a* showed *SULT1A3* and *SULT1A4* down- and upregulation respectively, but these two genes share exons with *SLX1A* and *SLX1B*, which were dysregulated in WT HEK cells. These two phenomena (read-through transcripts and exon sharing) raise the question of RNA-seq reliability for the detection of such transcripts. RT-qPCR experiments may be of interest to confirm, at least partly, some RNA-seq results.

### 4.4. PBX1 Transfection Gives Fluctuant Results in HEK Cells

Our approaches did not directly study the transactivation effects of the p.(Arg107Gln) mutant nor the subcellular localization of the protein. Previous functional studies targeting another variant in the same cell line (p.(Arg235Gln) introduced in WT HEK cell through plasmid transfection) led to discordant conclusions on the subcellular localization of the resulting protein [15,16]. These works also demonstrated impaired transactivation [15] or heterodimerization [16] properties of PBX1 mutant proteins. Nevertheless, the transactivation defects were discordant between a *Pbx1*-LoF murine cell line (obtained through CRISPR-Cas9 editing) and HEK cells [15]. Thus, the results observed in both studies and our work highlight the fact that HEK cells may not be the most accurate model to study PBX1 functions through plasmid transfection. In fact, HEK cells are probably derived from immature neuron-like renal cells rather than renal epithelial cells or fibroblasts [44,45]. HEK cells are pseudotriploid and some authors suggest a high instability of their karyotype after several passages or freezing cycles, especially for HEK 293T cells. Transfection-induced stress is also known to trigger genomic instability in these cell lines [45]. The naturally occurring overexpression of *PBX1* transcripts through *PBX1* genomic amplification was dismissed through aCGH. Genomic instability was highlighted in our cell lines, with WT and KD2 cells presenting with a few different genomic imbalances. These differences may also play a role in the fluctuant transfection results observed, despite the fact they seem to be expected variations in cultured HEK 293T cells. Finally, the dominant-negative effects observed in gastric cancer cells after the introduction of a missense mutation in *PBX1* may also rely on the fact that the mutant gene was introduced through infection techniques [31], leading to a more stable expression. Overall, CRISPR-Cas9 is an efficient tool to generate *PBX1*-KD clones derived from HEK cells, but plasmid transfection may not be the most efficient way to rescue the KD phenotype.

## 5. Conclusions

*PBX1* encodes a ubiquitous transcription factor, which can lead to severe phenotypes when disrupted. We reported here a third patient with a substitution of the Arg107 in one of the dimerization domains of the protein. CRISPR-Cas9 allowed us to generate a *PBX1*-KD clone with a cellular phenotype close to the mesenchymal defects observed in mouse embryos. We were not able to fully rescue the phenotype through WT or mutant *PBX1a* transfection, but the results suggest a disruption of splicing and DNA repair as suspected mechanisms in cells expressing the mutant *PBX1*. Further functional studies focusing on techniques other than plasmid transfection are needed to explore the effects of PBX1 Arg107 disruption.

## Figures and Tables

**Table 1:** *PBX1* isoforms expressed in transfected cells. WT HEK+WT/mutant: WT HEK cells transfected with WT or mutant *PBX1a* plasmid. Clone KD2 + WT/mutant: KD2 cells transfected with WT or mutant *PBX1a* plasmid. TPM: transcript per million.

PBX1 Isoform	WT HEK + WT TPM	WT HEK + Mutant TPM	Clone KD2 + WT TPM	Clone KD2 + Mutant TPM
NM_001204961 = **PBX1b**	1.60	2.56	0.22	1.10
NM_001204963	NA	NA	NA	NA
NM_001353130	0	0.31	0	0.16
NM_001353131 = **PBX1b**	1.91	1.79	0	1.07
NM_002585= XM_005245229 = **PBX1a**	1208.94	976.54	447.82	408.52
XM_005245228	NA	NA	NA	NA
XM_011509590	0	0	0	0.25
XM_011509591	0	0	0.46	0
XM_011509592	1.47	0.18	0.31	0
XM_017001395 (PBX1a ?)	2665.33	2435.52	1155.85	887.08

## Data Availability

The variant found in the patient was recorded in ClinVar database under the number SUB12167949. RNA-seq data were uploaded on the GEO site under the number GSE215967.

## Supplementary Materials

The following supporting information can be downloaded at: https://www.mdpi.com/article/10.3390/genes14020273/s1. Figure S1. Uncropped Western Blot of clones obtained after CRISPR-KO editing (a). Clone designed by an arrow was considered closest to a PBX1 KO (further called clone KD2 in main text). Clones in dashed boxes were re-analysed through WB (b). Arrowheads show PBX1a (black) and PBX1b (white) expected sizes. Figure S2. Sanger sequencing of clones selected in Figure S1 (dashed boxes, Figure S1a), showing a partially edited PBX1 exon 3 sequence: at least one PBX1 allele shows a frameshift, but the WT PBX1 sequence persists (HEK cells are triploid). Clone 7 is not edited. Clone 25 (sgRNA_2) died before DNA could be extracted for testing. Figure S3. Significant decrease in proliferation capacities of clone KD2 compared to WT HEK cells (WT) after 3 days of culture. Two counting methods were used to avoid cell pellet detachment bias that may occur with methylene blue count. Figure S4. Relative gene expression in WT HEK and KD2 cells lines after transfection. PBX1 WT: WT PBX1a plasmid, PBX1 320G>A: mutant PBX1a plasmid. *MEOX1* was not conserved for analyses due to a very low level of expression in both cell lines. Figure S5. Scatter plot of differentially expressed genes (DEGs) between samples. PBX1 is slightly less expressed in clones KD2 compared to WT HEK cells, whatever the transfected plasmid (upside panes, arrow). For a chosen cell type, PBX1 expression is similar between WT PBX1a-transfected and mutant PBX1a-transfected cells (downside panes, dashed arrow). HEK+WT/mutant: WT HEK cells transfected with WT or mutant PBX1a plasmid. Clone KD2+WT/mutant: KD2 cells transfected with WT or mutant PBX1a plasmid. Figure S6. PCA plot of RNA-seq samples. Figure S7. PBX1-320G>A-transfected cells express PBX1-mutated transcripts. IGV view of RNA sequence from PBX1 exon 3 in cells transfected with WT PBX1a-coding plasmid (WT) or PBX1a-320G>A-coding plasmid (mutant). Cells transfected with the mutated PBX1a form only express the PBX1a-320G>A form at the RNA level. HEK+WT/mutant: WT HEK cells transfected with WT or mutant PBX1a plasmid. Clone KD2+WT/mutant: KD2 cells transfected with WT or mutant PBX1a plasmid. Figure S8. Differential alternative splicing (as) events among groups. HEK+WT/mutant: WT HEK cells transfected with WT or mutant PBX1a plasmid. Clone KD2+WT/mutant: KD2 cells transfected with WT or mutant PBX1a plasmid. Skipped Exon (SE), Alternative 5’ Splicing Site (A5SS), Alternative 3’ Splicing Site (A3SS), Mutually exclusive exons (MXE), Retained Intron (RI). (a) Number (nb) of splicing variations among samples showed a decrease in splicing variations in clone KD2, (b) Due to these large variations in total splicing events among samples, comparison between types of splicing events was performed on relatives values using Z-test, with a threshold of 1.96 (α error rate of 5%, two tailed). % age: percentage. Figure S9. Uncropped Western-Blot from Figure 3 showing the PBX1 KD phenotype of clone KD2. Western Blot demonstrating the diminution of both PBX1a (black arrowhead) and PBX1b (white arrowhead) expression in PBX1 KD clone (clone KD2). WT HEK cells transfected with control siRNA (WT + si CTRL) or PBX1 siRNA (WT + si PBX1) served as negative and positive controls, respectively. WT: non-transfected HEK cells. Figure S10. Uncropped Western-Blot from Figure 4 assessing PBX1a overexpression after PBX1a-coding plasmid transfection. Uncropped WB assessing PBX1a overexpression (black arrowhead) in transfected HEK WT cells (HEK WT) and KD2 clones. White arrowhead: PBX1b. Figure S11. Comparison of aCGH profiles between WT HEK cells and clone KD2 before plasmid transfection. Table S1. DNA primers, siRNA and CRISPR guides sequences used in this study. Table S2. Summary of RNA-seq results in WT PBX1a- and mutant PBX1a-transfected cells. DEG: differentially expressed gene, DASE: differential alternative splicing events. Table S3. Relative quantification of PBX1a and PBX1b expression on Figure S1 Western-Blot.
