# Supplementary material for: Functional Assessment of a New PBX1 Variant in a 46,XY Fetus with Severe Syndromic Difference of Sexual Development through CRISPR-Cas9 Gene Editing
Source: Genes (Basel). 2023 Jan 20;14(2):273. doi: 10.3390/genes14020273 (PMC9956894; doi:10.3390/genes14020273)
Supplement: Supplementary file 1 [file genes-14-00273-s001.zip › Supplemeantary Table S2.pdf]

	HEK293T + WT *PBX1a* plasmid	HEK293T + mutant *PBX1a* plasmid	Clone KD2 + WT *PBX1a* plasmid	Clone KD2 + mutant *PBX1a* plasmid
HEK293T + WT *PBX1a* plasmid		23 DEGs (DNA repair, telomeres maintenance, oocyte differentiation)<br><br>44 DASE	2118 DEGs (transcription regulation, extracellular matrix organization, primary cilium proteins, signal transduction pathways)<br><br>461 DASE	Not comparable
HEK293T + mutant *PBX1a* plasmid	23 DEGs (DNA repair, telomeres maintenance, oocyte differentiation)<br><br>44 DASE		Not comparable	1985 DEGs (transcription regulation, signal transduction pathways)
Clone KD2 + WT *PBX1a* plasmid	2118 DEGs (transcription regulation, extracellular matrix organization, primary cilium proteins, signal transduction pathways)<br><br>461 DASE	Not comparable		9 DEGs (dopamine and steroid metabolism and cytokine production)<br><br>18 DASE
Clone KD2 + mutant *PBX1a* plasmid	Not comparable	1985 DEGs (transcription regulation, signal transduction pathways)	10 DEGs (dopamine and steroid metabolism and cytokine production)<br><br>18 DASE	

Supplementary Table S2 – Summary of RNA-seq results in WT PBX1a- and mutant PBX1a-transfected cells.

DEG: differentially expressed gene, DASE: differential alternative splicing events.
