# Supplementary figures and images for: Functional Assessment of a New PBX1 Variant in a 46,XY Fetus with Severe Syndromic Difference of Sexual Development through CRISPR-Cas9 Gene Editing
Source: Genes (Basel). 2023 Jan 20;14(2):273. doi: 10.3390/genes14020273 (PMC9956894; doi:10.3390/genes14020273)

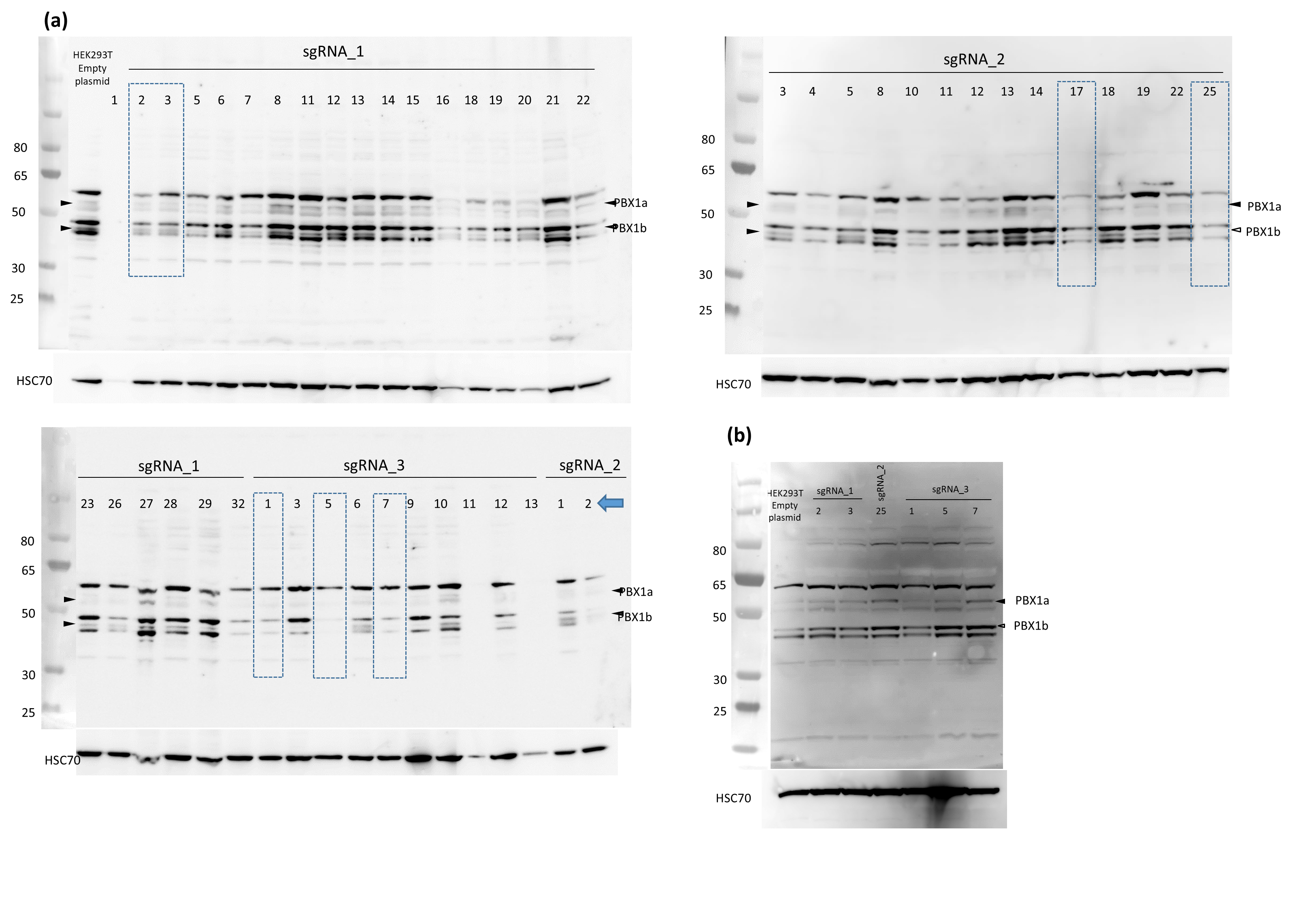

Supplement: Supplementary file 1 [file genes-14-00273-s001.zip › Figure S1.png]

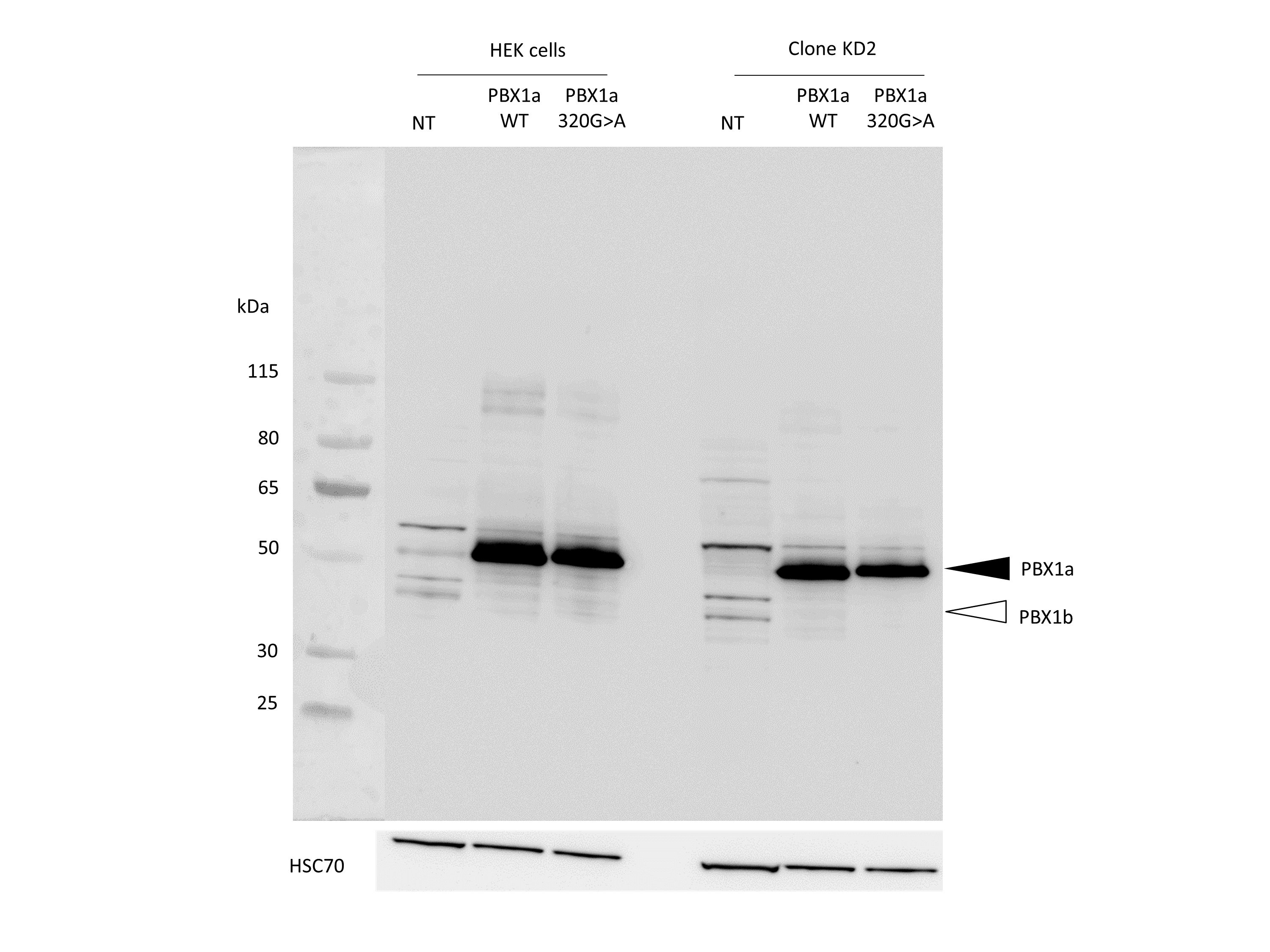

Supplement: Supplementary file 1 [file genes-14-00273-s001.zip › Figure S10.png]

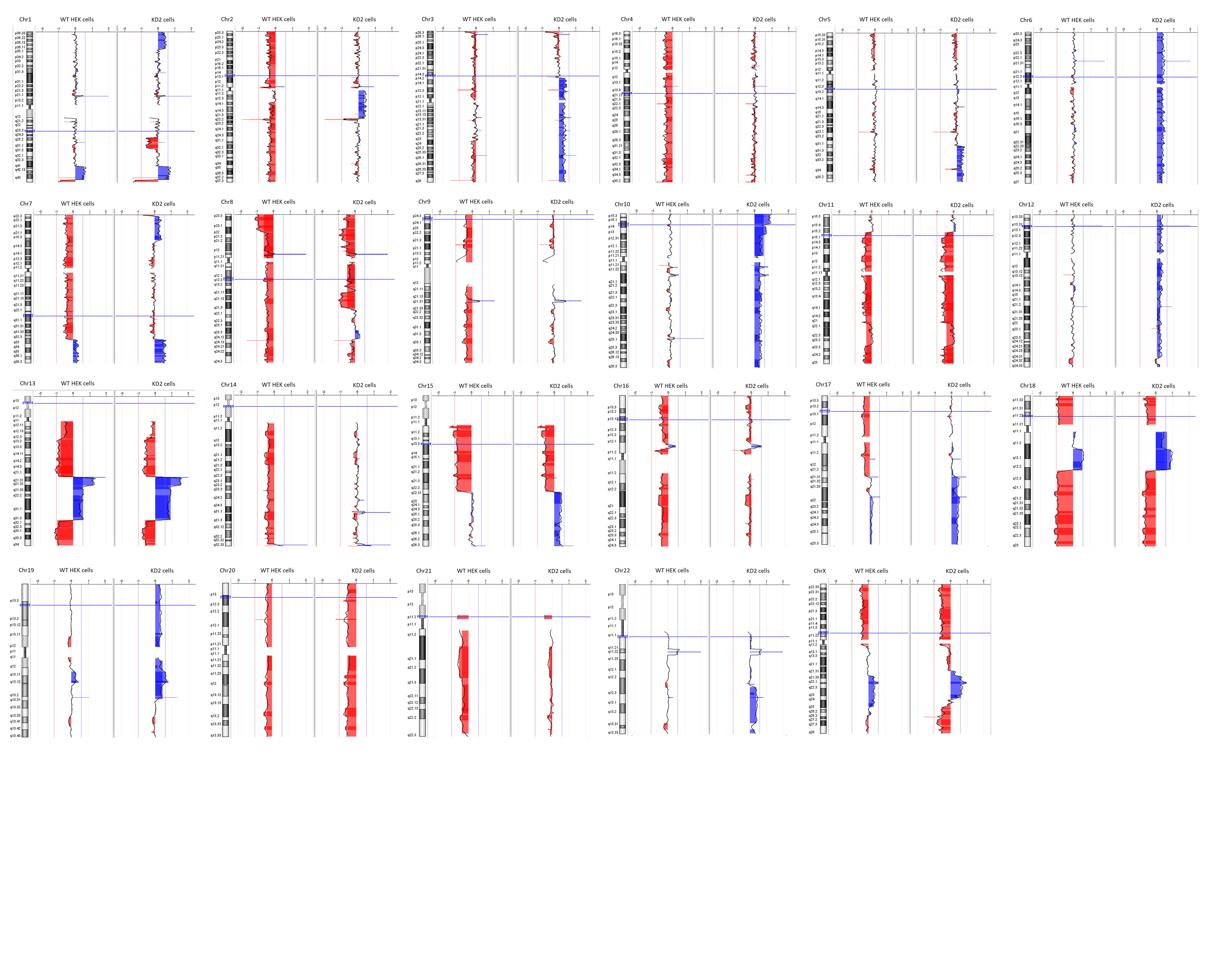

Supplement: Supplementary file 1 [file genes-14-00273-s001.zip › Figure S11.png]

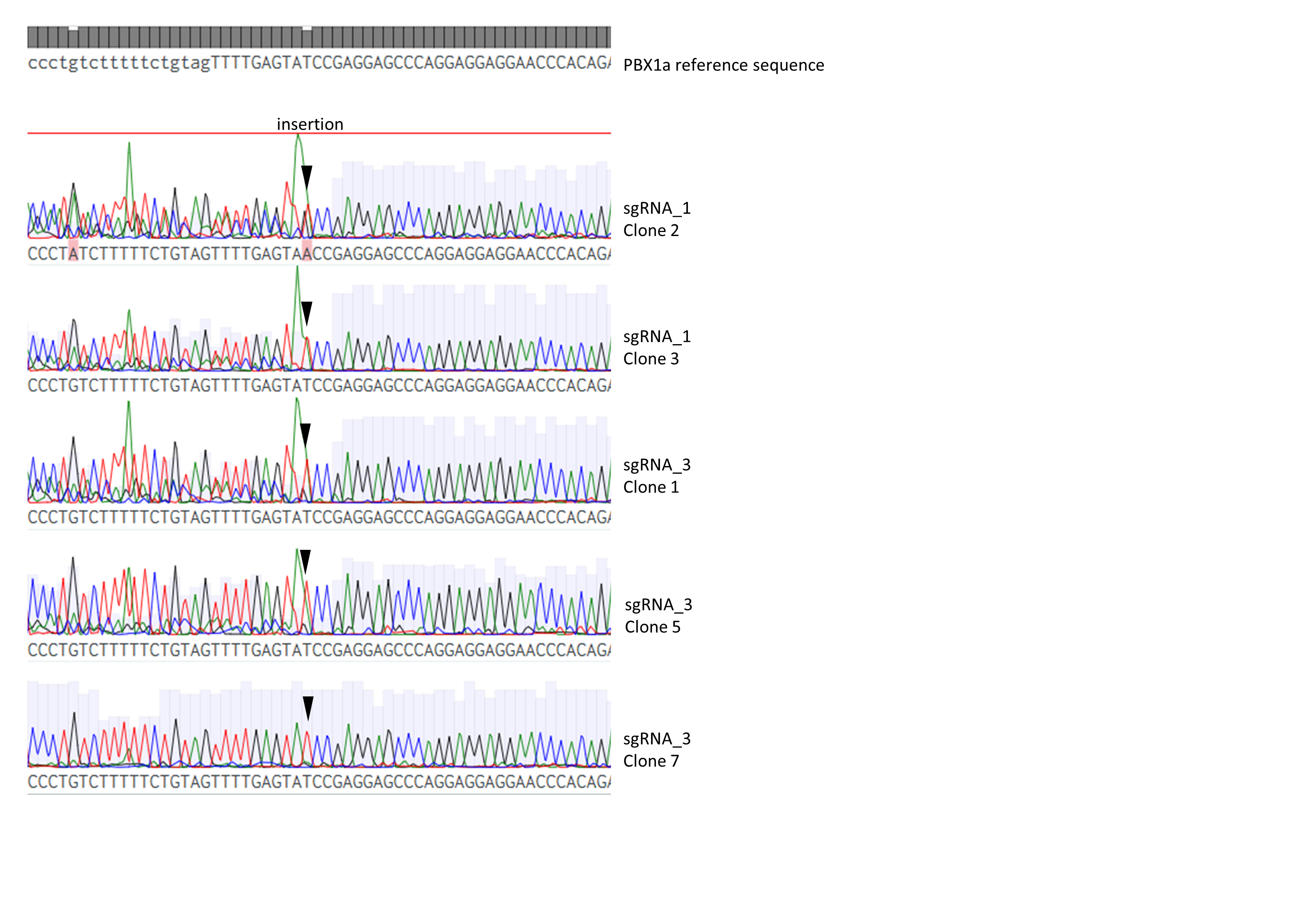

Supplement: Supplementary file 1 [file genes-14-00273-s001.zip › Figure S2.png]

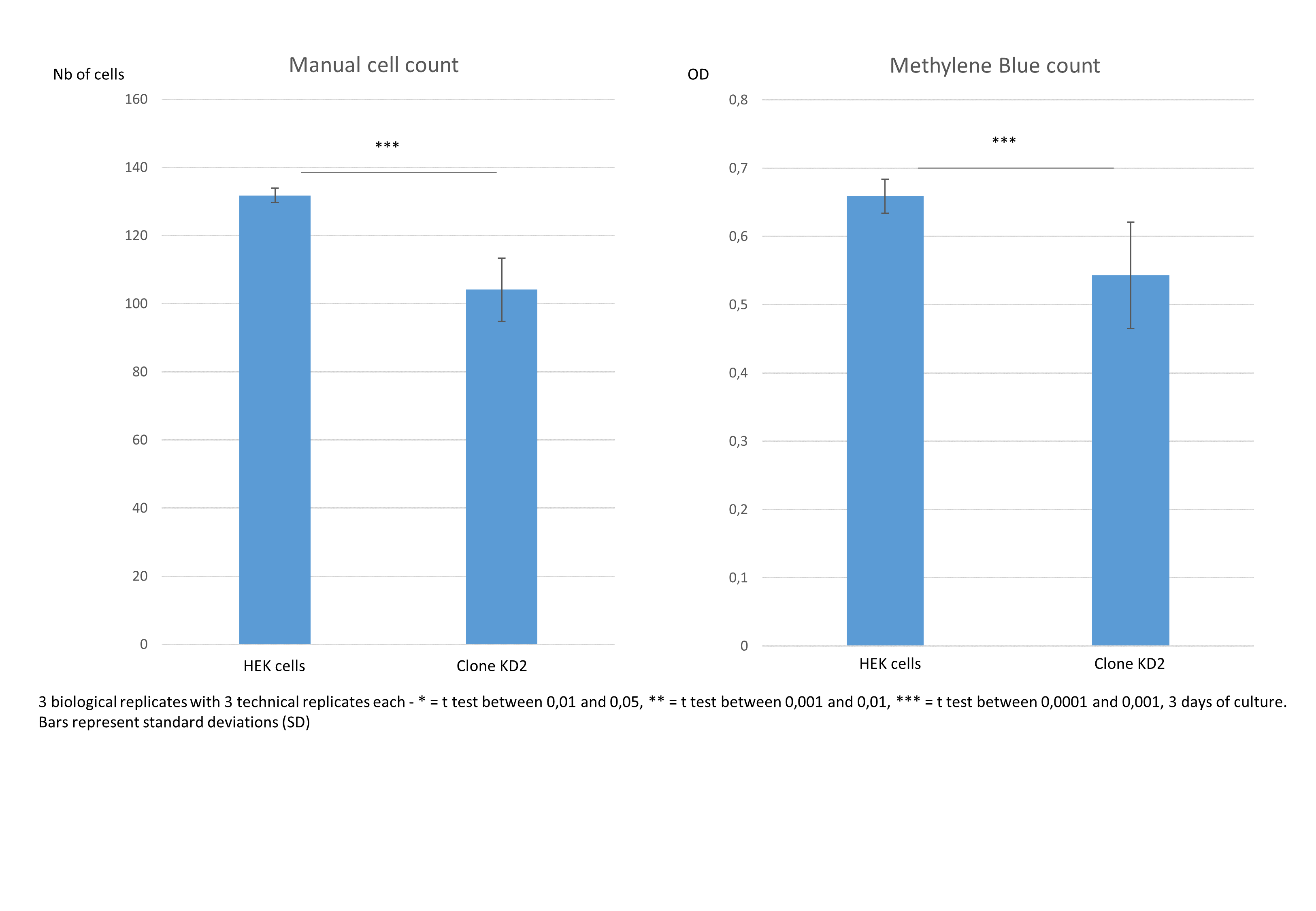

Supplement: Supplementary file 1 [file genes-14-00273-s001.zip › Figure S3.png]

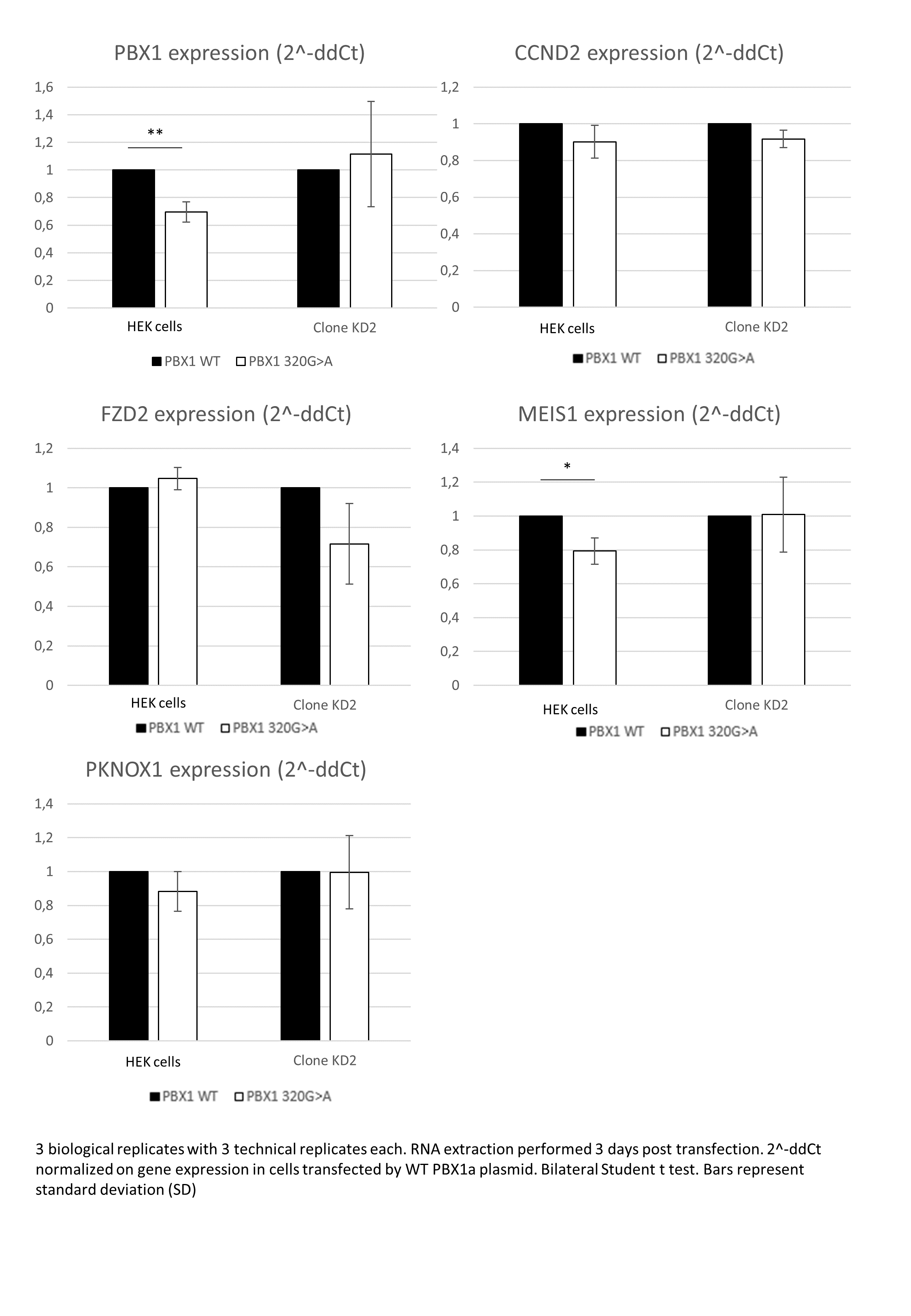

Supplement: Supplementary file 1 [file genes-14-00273-s001.zip › Figure S4.png]

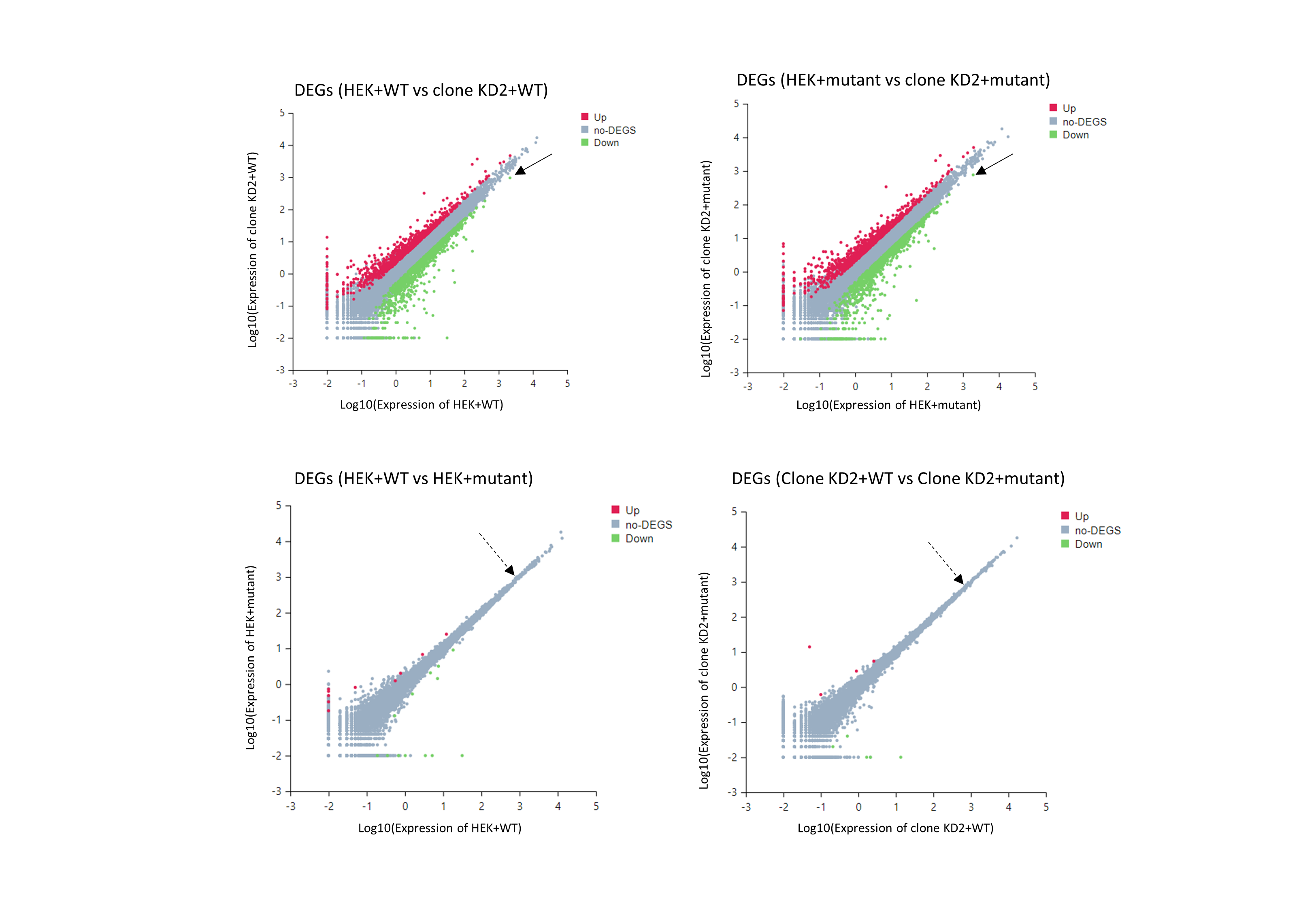

Supplement: Supplementary file 1 [file genes-14-00273-s001.zip › Figure S5.png]

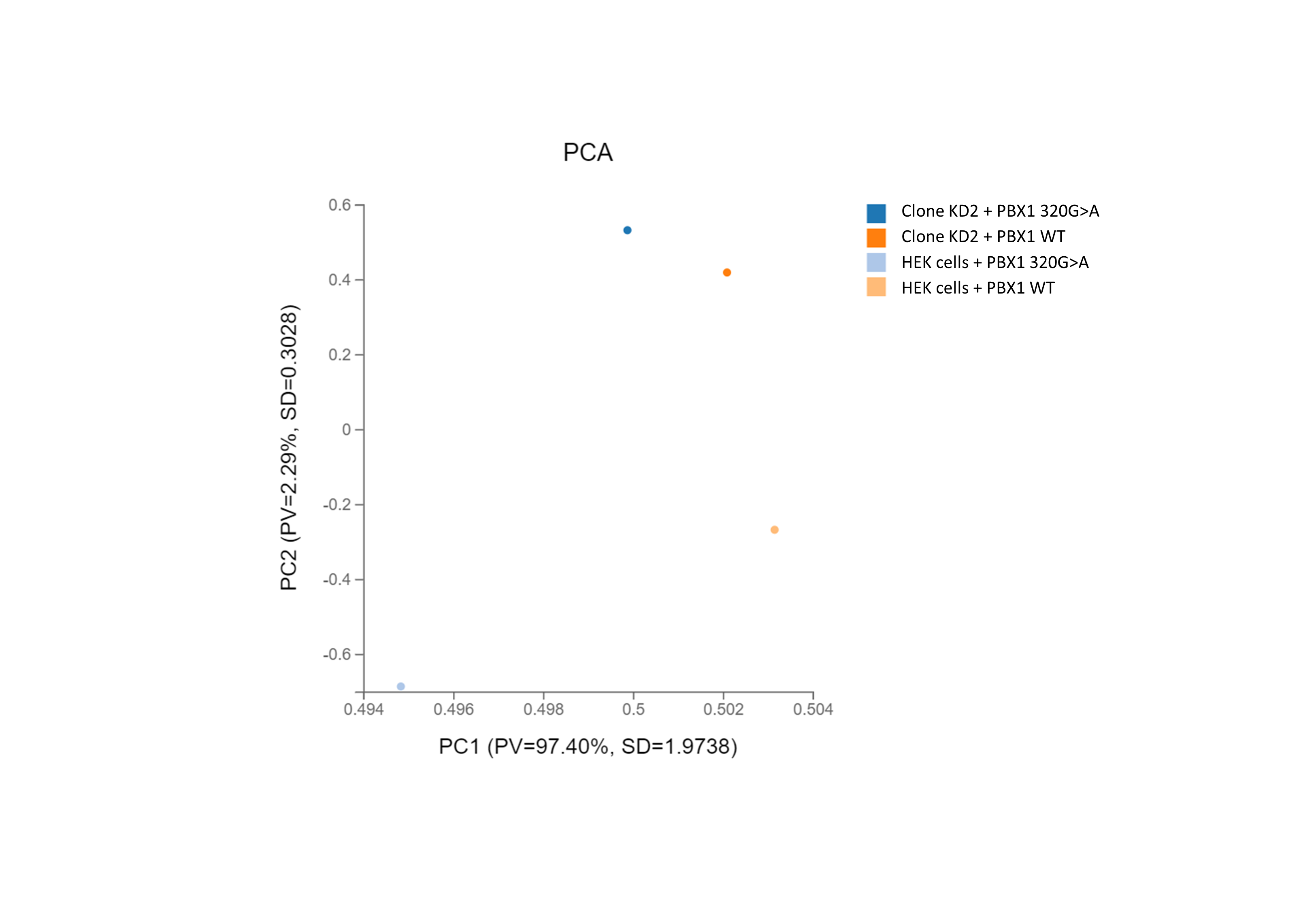

Supplement: Supplementary file 1 [file genes-14-00273-s001.zip › Figure S6.png]

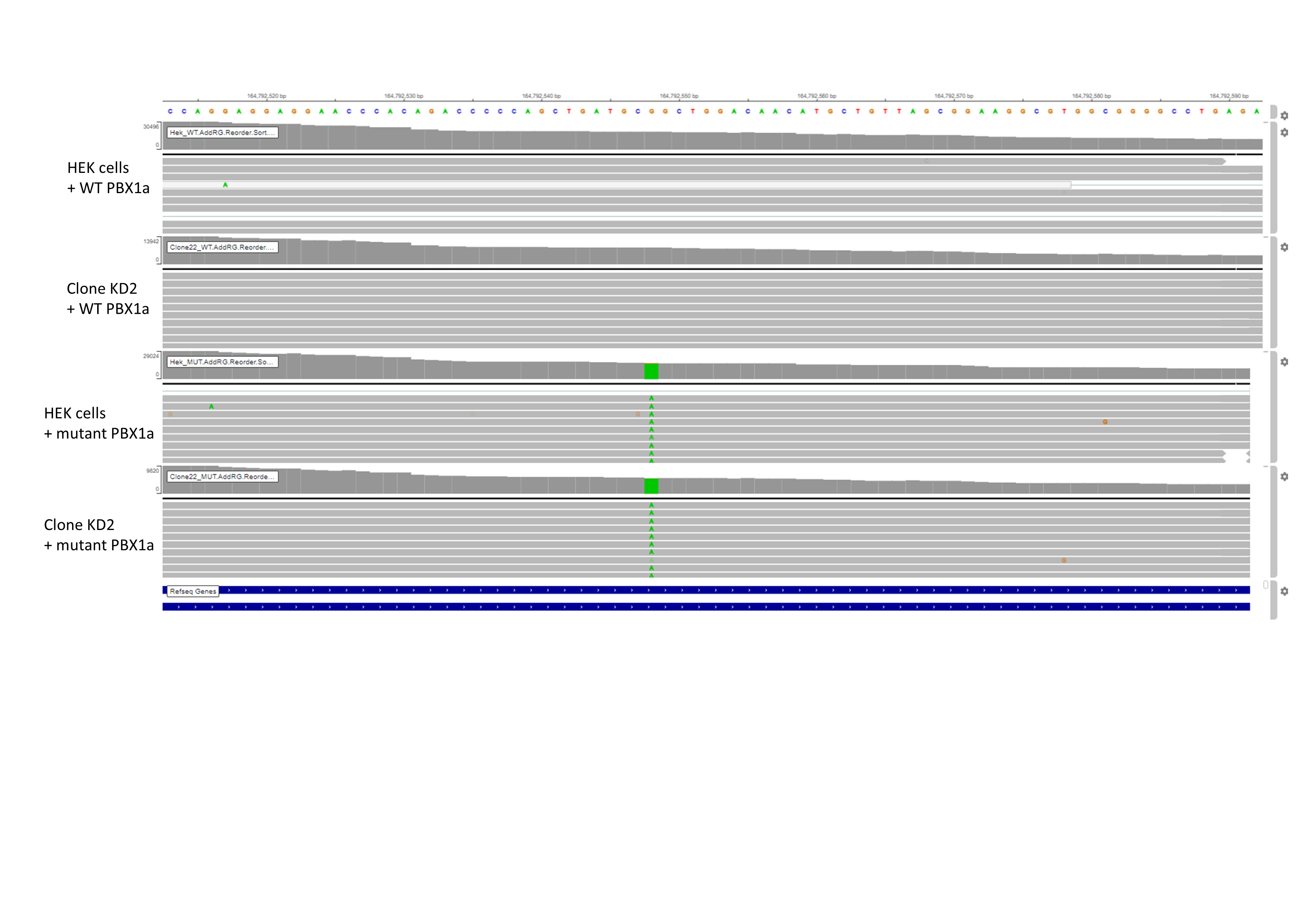

Supplement: Supplementary file 1 [file genes-14-00273-s001.zip › Figure S7.png]

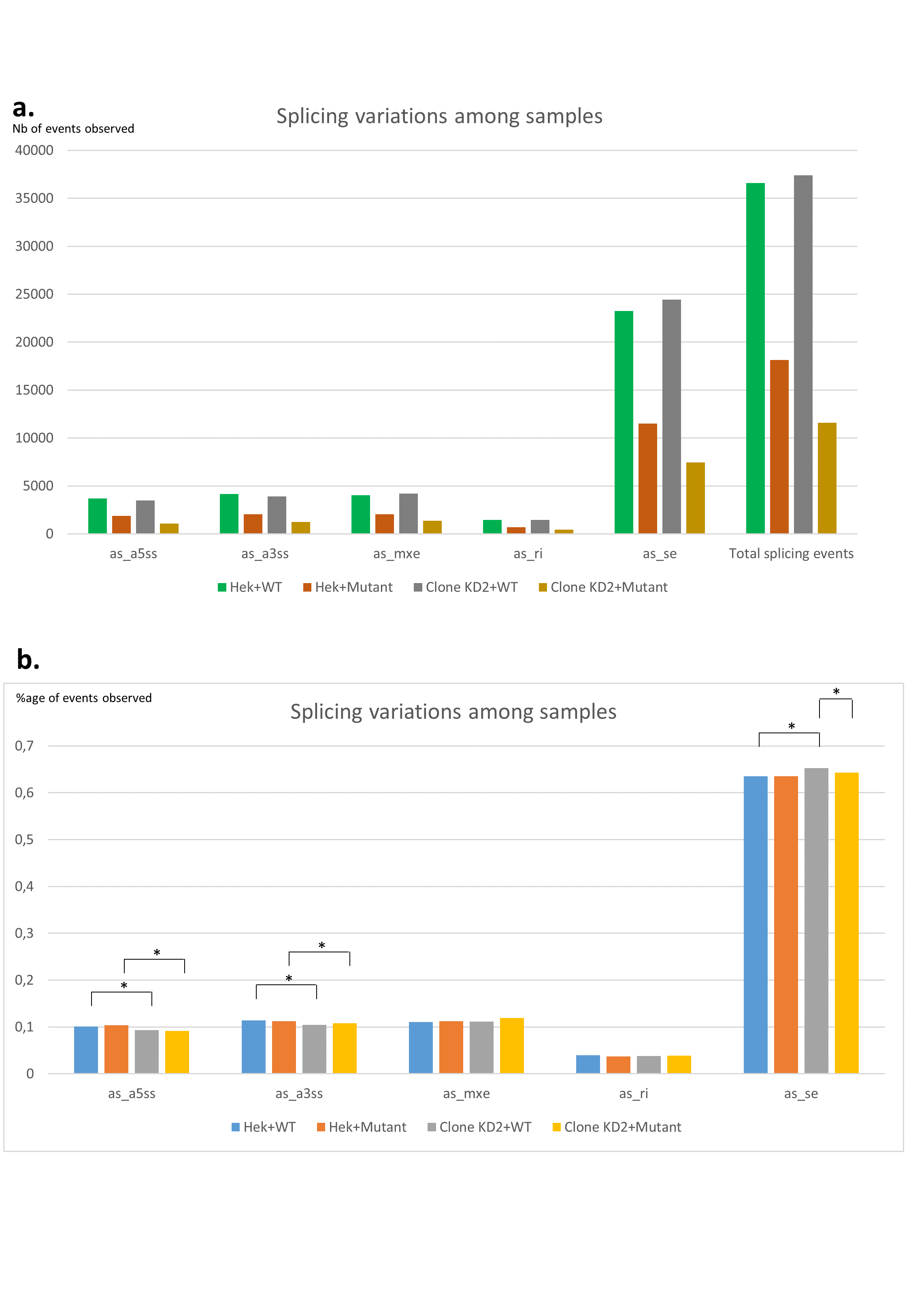

Supplement: Supplementary file 1 [file genes-14-00273-s001.zip › Figure S8.png]

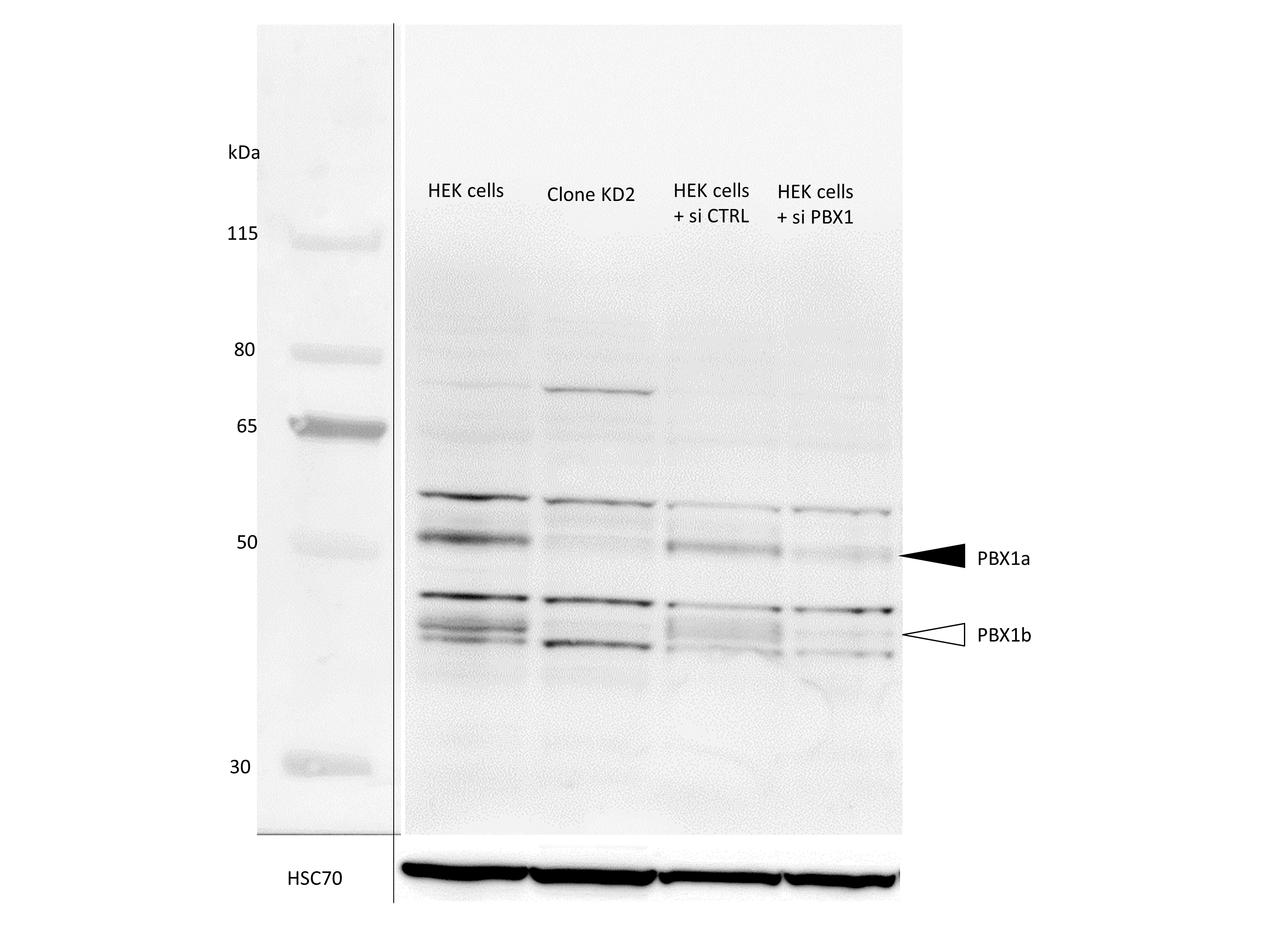

Supplement: Supplementary file 1 [file genes-14-00273-s001.zip › Figure S9.png]
